# Frequent Activation of Notch Signaling Pathway in Colorectal Cancers and Its Implication in Patient Survival Outcome

**DOI:** 10.1155/2020/6768942

**Published:** 2020-03-09

**Authors:** Jilani Purusottapatnam Shaik, Ibrahim O. Alanazi, Akbar Ali Khan Pathan, Narasimha Reddy Parine, Majid A. Almadi, Nahla A. Azzam, Abdulrahman M. Aljebreen, Othman Alharbi, Mohammad Saud Alanazi, Zahid Khan

**Affiliations:** ^1^Genome Research Chair, Department of Biochemistry, College of Science, King Saud University, Riyadh, Saudi Arabia; ^2^National Center for Biotechnology, King Abdulaziz City for Science and Technology, Riyadh, Saudi Arabia; ^3^College of Medicine, King Saud University, Riyadh, Saudi Arabia; ^4^Division of Gastroenterology, King Khalid University Hospital, King Saud University, Riyadh, Saudi Arabia

## Abstract

Colorectal cancer is a major health concern as it ranks third in incidence and second major cause of cancer-related deaths worldwide. A leading cause of treatment failure has been attributed to cancer stem cells that can invariably resist existing chemotherapeutic regimens. Notch signaling pathway has been involved in the maintenance of stem cells besides being crucial in cell fate decision and embryonic development. This pathway has also been implicated in several human malignancies including colorectal cancer. We investigated mRNA expression of four Notch receptors (Notch1–4), five ligands (Jag1, Jag2, Dll1, Dll3, and Dll4), and four target genes (Hes1, Hes5, Hey1, and Hey2) using highly specific TaqMan gene expression assays in colorectal adenomas and cancers. Upregulated expression of Notch receptors ranged between 29 and 73% in colorectal cancers and between 11 and 56% in adenomas. Expression of Notch3 and Notch4 receptors was significantly higher in colorectal cancers compared to normal and adenoma tissues. The Jagged and Delta-like ligands were overexpressed between 25 and 52% in colorectal cancers, while in adenomas, it ranged between 0 and 33%. Combining the data for upregulation of receptors and ligands suggests that 86% colorectal cancers and 56% adenomas exhibited overexpression of Notch pathway genes in our cohort. Notch target genes were upregulated between 24 and 33% in colorectal cancers and between 11 and 22% in adenomas. Collating upregulation of Notch receptors and ligands with the target genes showed concordance in 58% colorectal tumors. Additionally, we evaluated expression of Notch receptors, ligands, and target genes with prognosis using the TCGA mRNA expression dataset. Patients overexpressing Notch3, Notch4, and Hey1 had significantly poorer overall survival relative to those having lower levels of these genes. Taken together, Notch signaling components are aberrantly overexpressed in colorectal tumors, and development of therapeutics targeting the Notch pathway may prove to be beneficial in the management of colorectal cancers.

## 1. Introduction

Colorectal cancer (CRC) is the second most common cancer in women and ranks third in men worldwide [1]. CRC is a group that includes cancers of the colon, rectum, and anus. For both sexes combined, CRC is the third most commonly diagnosed cancer representing 10.2% of the total cases and second leading cause of cancer-related death (9.2%) worldwide in 2018 [1]. In Saudi Arabia, it is the most common cancer in males (19.6%), while in female, it ranks number three (9.5%). The cancer-related mortality in Saudi Arabia is highest for patients with CRC accounting for 15.2% of all deaths due to cancer [2].

Distant metastasis is the main cause of colorectal cancer-related death [3]. Despite several advances in early diagnosis and treatments that include chemotherapy, immunotherapies, antiangiogenics, and surgical treatment, a significant percentage of early-stage cases will develop metastases and eventually die. This resistance to therapy eventually leading to fatality has been mainly attributed to cancer stem cells (CSCs). Understating the mechanism about how these cells are able to resist existing treatments may lead to the development of new therapies targeting CSCs [4].

Several molecular pathways are known to be involved in the functioning of CSCs including Wnt, Hedgehog, and Notch signaling [5]. Notch is a highly conserved signaling pathway that plays an important role in embryogenesis and cell fate determination whose genes were discovered in 1917 as a result of abnormal formation of notches in the wings of Drosophila melanogaster [6, 7]. Besides implication in several developmental defects in humans, this pathway has been implicated in the pathogenesis of leukemia and several other cancers including colorectal cancers [8–10]. Aberrantly activated Notch signaling plays a crucial role in carcinogenesis and progression of various human malignancies including the breast [11] and colorectal cancer [12]. The overexpressions of Notch signaling components are correlated with CRC progression and metastasis [13]. However, the involvement of Notch signaling in early-stage CRC has not been well understood, and only few studies have been undertaken to investigate its role [14, 15]. Interestingly, besides oncogenic role associated with tumor progression and metastasis, Notch also functions as tumor suppressor [16]. In mammals, the Notch pathway consists of five ligands and four receptors, which are single-pass transmembrane proteins. The five ligands are members of the Delta/Serrate/LAG-2 (DSL) family of proteins and include Delta-like-1 (Dll-1), Delta-like-3 (Dll-3), Delta-like-4 (Dll-4), Jagged-1, and Jagged-2, while receptors include Notch1, Notch2, Notch3, and Notch4 [17].

Activation of Notch pathway occurs via the binding of Notch ligands with their receptors. This activation leads to a proteolytic cleavage of the Notch protein via disintegrin and metalloproteinase domain-containing protein 17 (ADAM17) and disintegrin and metalloproteinase domain-containing protein 10 (ADAM10) in the extracellular domain followed by the *γ*-secretase enzyme complex cleaving the transmembrane domain. As a result, Notch intracellular domain (NICD) is released and translocated to the nucleus forming a complex with transcription factor CSL and transcriptional coactivators including mastermind-like polypeptides (MAML) and recombination signal binding protein for immunoglobulin kappa J region (RBPj). This interaction leads to the activation of various target genes associated with cell survival, differentiation, cell growth, and tumorigenesis [18].

In this study, we investigated the involvement of the Notch signaling pathway in colorectal adenomas and cancers arising in Saudi Arabian patients by examining the expression of Notch receptors, ligands, and target genes. Additionally, using the mRNA expression data from TCGA, we studied the correlation of Notch pathway gene expression with the survival outcome of colorectal cancer patients.

## 2. Materials and Methods

### 2.1. Patient Samples

Tissue biopsies from colorectal cancers (*n* = 66), adenomas (*n* = 9), and corresponding adjacent normal were obtained from the Gastroenterology division of King Khalid University Hospital (KKUH), Riyadh, Saudi Arabia. The specimens were collected and stored in RNA stabilizing reagent (Qiagen, Germany) at 4°C for 24 h and then at −80°C till the time of RNA extraction. Samples were obtained from the patients prior to receiving chemotherapy or radiation therapy. The Ethics Committee of KKUH approved the study, and written informed consent from the patients was obtained.

### 2.2. RNA Extraction and cDNA Synthesis

Tissues were homogenized in a Dispomix tube by Dispomix drive (Medic Tools, AG, Zug, Switzerland). AllPrep DNA/RNA Mini Kit (Qiagen, Germany) was used to extract total RNA as per the manufacturer. On-column DNaseI treatment was performed using RNase-Free DNase Set (Qiagen, Germany). RNA integrity was determined by RNA 6000 Nano kit and 2100 Bioanalyzer system (Agilent Technologies Inc., California, USA). RNA quantitation was performed on NanoDrop™ 8000 Spectrophotometer (Thermo Fisher Scientific). RNAs were reverse-transcribed to obtain cDNA using high-capacity cDNA reverse transcription kit (Thermo Fisher Scientific) according to the manufacturer and stored at −20°C.

### 2.3. Real-Time RT-PCR

Real-time quantitative PCR reactions were performed in triplicate for each gene in a final volume of 10 *μ*l. Each reaction contains 0.5 *μ*l of 20X TaqMan gene expression assay, 5.0 *μ*l of 2X TaqMan gene expression master mix, 2.0 *μ*l cDNA, and 2.5 *μ*l nuclease-free water. Amplification was performed using QuantStudio™ 7 Flex Real-Time PCR System (Applied Biosystems) at the following reaction conditions: AmpliTaq Gold DNA Polymerase hot start activation at 95°C for 30 s and 40 cycles at 95°C for 15 s and 60°C for 60 s. TaqMan assay IDs for the Notch pathway genes and GAPDH that was used as housekeeping gene are shown in Table 1. mRNA expression fold change in colorectal cancers and adenomas relative to adjacent normal tissues was calculated using the 2^−ΔΔCt^ method. Additionally, we also calculated expression of each gene by using the 2^ΔCt^ method using GAPDH as reference gene in normal, adenoma, and cancer tissues.

### 2.4. Kaplan–Meier Analysis

To evaluate the effect of Notch pathway gene expression on the prognosis of colon adenocarcinoma patients, Kaplan–Meier survival analysis was performed using mRNA expression data from studies performed by The Cancer Genome Atlas (TCGA) obtained from OncoLnc, http://www.oncolnc.org. Patients were sorted according to gene expression levels, and bottom third (*n* = 145) versus top third (*n* = 145) expressers were compared; hence, lower and upper percentiles were set as 33 : 33 for the survival curve analysis.

### 2.5. Statistical Analysis

Statistical significance for the expression of Notch pathway components between normal, adenoma, and colorectal cancer was determined using the two-tailed Student's *t*-test. The survival rate was calculated using Kaplan–Meier estimator and was assessed by the log-rank test on OncoLnc (http://www.oncolnc.org/), an online analysis tool. A *p* value <0.05 was considered to indicate statistical significance.

## 3. Results

### 3.1. Notch Signaling Pathway Activation in Colorectal Adenomas and Cancers

**Figure 1:**
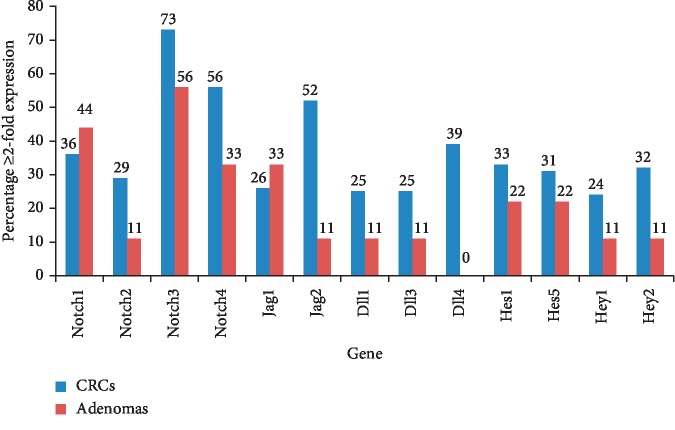
Expression of Notch signaling genes. mRNA of the indicated genes was overexpressed ≥2-fold in colorectal adenomas and cancers relative to the adjacent normal tissues determined using the 2^−ΔΔCt^ method. The numbers on top of the bars indicate percentages of adenomas and colorectal cancers exhibiting ≥2-fold expression.

We examined relative mRNA expression of four Notch receptors (Notch1-4), two Jagged ligands (Jag1 and 2), three Delta-like ligands (Dll1, 3, and 4), and four target genes (Hes1, Hes5, Hey1, and Hey2) in order to determine activation of the Notch signaling pathway in colorectal adenomas (*n* = 9) and cancers (*n* = 66). Percentages of adenomas and CRCs that exhibited overexpression ≥2-fold compared to the adjacent normal tissues for each gene are shown in Figure 1. Among the receptors, aberrant Notch3 and Notch4 overexpressions were observed in high percentage of colorectal tumors seen in 44/66 (73%) and 37/66 (56%) cases, respectively. Notch1 and Notch2 were overexpressed ≥2-fold in 36% and 29% of CRCs, respectively. Jag2 was the most frequently observed ligand that was overexpressed in 52%, while a higher expression of Jag1, Dll1, Dll3, and Dll4 was found in 26%, 25%, 25%, and 39% colorectal cancers, respectively. Comparatively, in adenomas, ≥2-fold Notch3 expression was detected in 56% followed by Notch1 (44%), Notch4 (33%), and Notch2 (11%), while for ligand Jag1, mRNA was overexpressed in 33%, Jag2, Dll1, and Dll3 in 11%, whereas Dll4 was not expressed in adenomas.

Activation of Notch signaling can occur as a result of abnormal overexpression of either of the Notch receptors or ligands. Combining the data for the deregulated expression of these components suggests that the pathway activation could be detected in 86% (57/66) of CRCs and 56% (5/9) of adenomas in our cohort. The difference between activation of the Notch pathway in adenomas versus colorectal cancers was found to be statistically significant (*p*=0.0431).

Notch target genes, Hes1, Hes5, Hey1, and Hey2, were upregulated ≥2-fold in 33%, 31%, 24%, and 32% of CRCs, respectively, whereas in adenomas, they were overexpressed in 22%, 22%, 11%, and 11%, respectively. Collating upregulation of Notch receptors and/or ligands that were seen in 57 CRCs with Notch target genes revealed that one or more targets were upregulated in 58% (33/57) colorectal tumors. Similar concordances were also observed in adenomas where the target genes were overexpressed in 60% (3/5) cases in which Notch receptors or ligands were upregulated.

**Figure 2:**
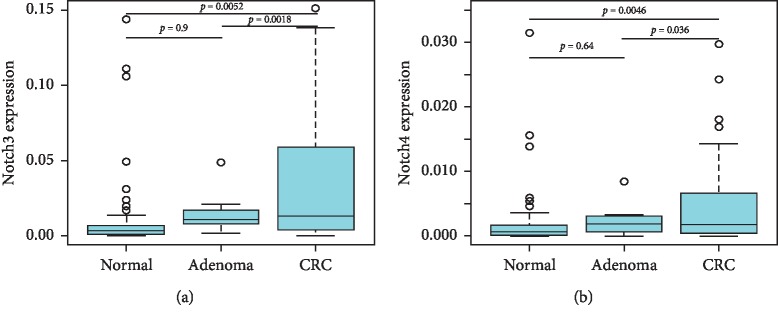
mRNA expression of Notch3 (a) and Notch4 (b) in normal, adenoma, and colorectal cancer tissues by the 2^ΔCt^ method using GAPDH as reference gene. A *p* value <0.05 was considered as significant. CRC, colorectal cancer.

**Figure 3:**
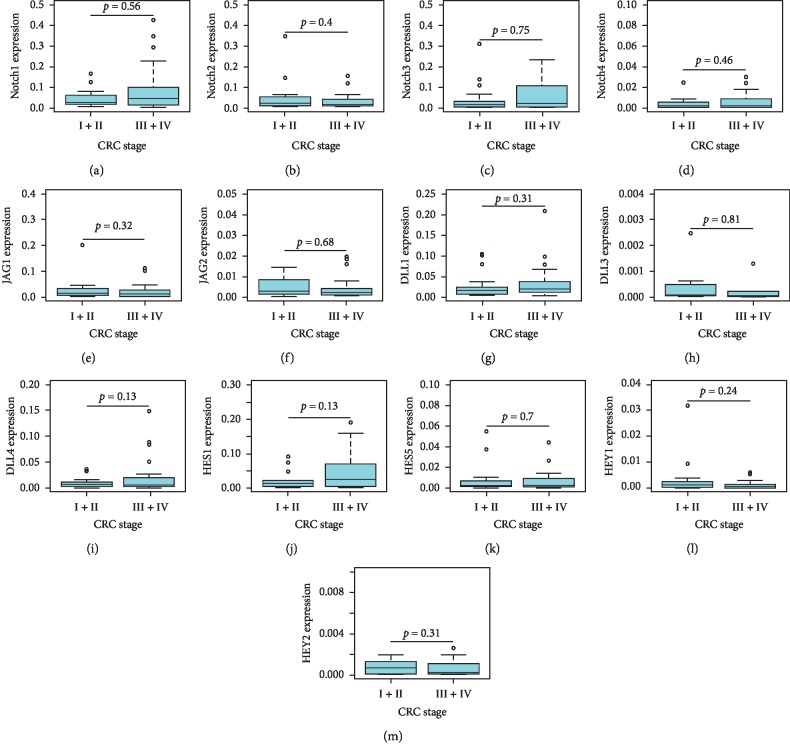
mRNA expression of the indicated Notch receptors (a–d), Notch ligands (e–i), and Notch target genes (j–m) in early-stage (I + II) and late-stage (III + IV) colorectal cancer tissues by the 2^ΔCt^ method using GAPDH as reference gene. A *p* value <0.05 was considered as significant. CRC, colorectal cancer.

Besides calculating fold-change expression of Notch pathway genes in adenomas and colorectal cancers relative to the adjacent normal tissues, we further calculated expression of Notch3 and Notch4 by the ΔCt method using GAPDH as reference gene in normal, adenoma, and cancer tissues. Notch3 and Notch4 expressions were significantly higher in colorectal cancers compared to normal and adenoma tissues (Notch3: normal vs. CRC, *p*=0.0052, adenoma vs CRC, *p*=0.0018; Notch4; normal vs. CRC, *p*=0.0046, adenoma vs. CRC, *p*=0.036) (Figure 2). Furthermore, we compared Notch pathway gene expression based on clinical stages of the 43 colorectal tumors for which TNM staging information was available. None of the examined genes showed significant differences in their expression in early-stage (Stages I and II) compared to late-stage (Stages III and IV) cancers (Figure 3).

### 3.2. Association of Notch Pathway Gene Expression with Prognosis

**Figure 4:**
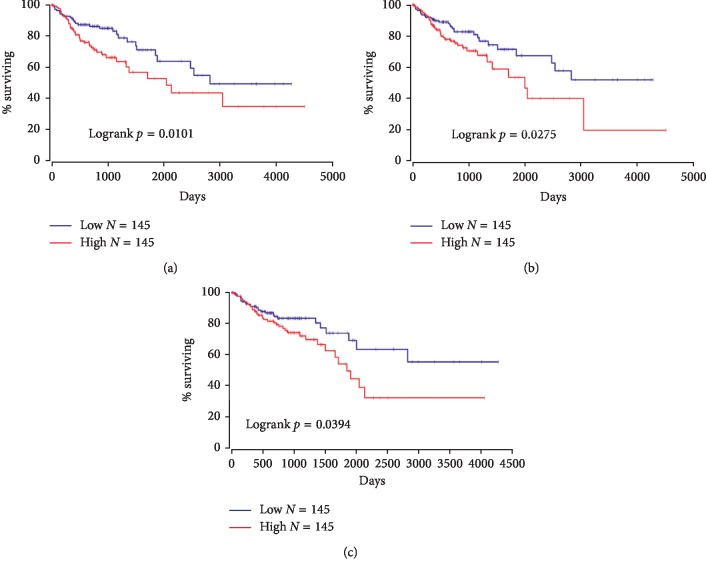
Kaplan–Meier survival curve. Overall survival outcome in colon adenocarcinoma patients expressing low and high mRNA levels of Notch3, (a) Notch4, (b) and Hey1 (c). A *p* value <0.05 was considered as significant.

The survival correlation of colon adenocarcinoma patients with Notch pathway gene mRNA expression was obtained from OncoLnc, a web-based tool publicly available at http://www.oncolnc.org that contains survival data of 21 cancers from studies performed by The Cancer Genome Atlas (TCGA). Of the 13 Notch pathway genes that we investigated, mRNA expression of Notch3, Notch4, and Hey1 was significantly associated with survival. Colon adenocarcinoma patients with a higher mRNA expression of Notch3, Notch4, and Hey1 presented significantly a shorter overall survival compared to those with low expression (Figure 4).

## 4. Discussion

Notch signaling pathway plays an important role in controlling the stability between cell proliferation, apoptosis, and differentiation. Therefore, it has been proposed that this signaling might be responsible for human tumor development and progression as it has been commonly altered in several malignancies [19]. As a result, Notch signaling pathway proteins can be exploited for novel cancer therapeutic targets [20]. CRC has frequent genetic and physiological alterations that may affect proliferation and cell survival, and Notch signaling is known to be one of the common alterations. This signaling has the ability to control the homeostatic self-renewal of intestinal epithelium as well as its tumorigenic transformation as both these processes are extraordinarily symmetrical [21]. In the control of intestinal cell fate, for instance, the decision between secretary and adsorptive cells can be regulated via the expression of Hes1, one of the Notch target genes [21]. Thus, its deregulated expression may lead to abnormality in the intestine. Our data show that Hes1 is upregulated ≥2-fold in 33% of CRCs while was overexpressed in 22% in adenomas. Several investigations indicate that overexpression of Notch1, Jag1, and Jag2 was found in human intestinal adenomas, implying that elevated Notch signaling might be responsible for colorectal cancer initiation [22–24]. Apart from activating mutations of Notch1 in T-cell acute lymphoblastic leukemia (T-ALL), somatic mutations in Notch1, 2, 3, and 4 genes in common solid cancers are rare [8, 25]. However, using datasets from the Cancer Cell Line Encyclopedia that contains mutational status of 1651 genes across 905 cancer cell lines and datasets on the cBioPortal for more than 2900 primary tumors, Mutvei et al. showed that the mutation frequency of Notch receptor and ligand genes are similar to that of established oncogenes but are higher in cell lines than in associated primary tumors [26].

Abnormal overexpression of Notch receptors or ligands leads to the activation of Notch signaling in CRC. For instance, our data showed that Notch1 was overexpressed ≥2-fold in 36% of CRCs, while Jag2 was found to be the predominant ligand that was overexpressed in 52% compared with Jag1 upregulation in 26% of CRCs. Several studies have demonstrated antitumor efficacy by therapeutically targeting the Notch signaling pathway. Concurrently targeting Notch and Erbb2 pathways in metastatic colorectal cancers that were resistant to cetuximab resulted in antitumor response, suggesting dependence of tumor on Notch and Erbb2 [27]. Additionally, targeting Dll4-Notch signaling using anti-Dll4 either alone or in combination with irinotecan showed efficacy in mutant KRAS colorectal xenograft tumors that were insensitive to cetuximab [28]. Anti-Dll4 antibody has also shown to inhibit breast cancer growth [29]. Furthermore, in KRAS-mutant lung adenocarcinoma, combined inhibition of DDR1 and Notch signaling resulted in regression of patient-derived xenografts with an efficacy comparable to standard chemotherapy [30]. Mullendore and coauthors observed overexpression of Notch ligands, predominantly Jag2 and Dll4 that were observed in 90% and 50% pancreatic cancer cell lines, respectively. They suggested that sustained activation of Notch signaling is required for the maintenance of pancreatic cancer as inhibition of Notch signaling abrogated anchorage-independent growth of pancreatic cancer cells [31]. Gamma-secretase cleaved activated Notch1 and Notch3 proteins have been detected in majority of breast cancer cell lines. Downregulation of Notch3 but not Notch1 considerably suppressed proliferation and induced apoptosis of the Erbb2-negative breast cancer cell lines implicating Notch3-mediated signaling in the proliferation of these cells [32]. Bernasconi-Elias and colleagues generated Notch3 inhibitory antibodies that exhibited strong antileukemic activity in cell lines as well as tumor xenografts with Notch3 activating mutations [33]. Notch3 gene amplification resulting in significant protein overexpression has been linked with proliferation and survival of ovarian carcinomas as inactivation of Notch3 suppressed proliferation and promoted apoptosis in cell lines overexpressing Notch3 [34]. Rallis G et al. showed that the overexpression of Notch3 in CRC patients was associated with bad outcomes, with about 2.5 times reduced disease-free survival (DFS) and overall survival (OS) [35]. Another study also confirmed a link between high levels of Notch3 and aggressive malignant colorectal cancer cell phenotype [36] and tumor recurrence after surgical resection of CRC [37, 38]. Our data indicate that aberrant Notch3 overexpression was observed in high proportion of colorectal tumors seen in 73% cases, and its expression was drastically associated with poor survival (*p*=0.0101). In view of these observations, inactivation of Notch3 signaling may prove beneficial in the therapeutic outcome of colorectal cancers.

Guiyang Wu and colleagues point out that the upregulation of Notch4 was significant in cancer tissues and liver metastases compared with adjacent normal tissues. Also, they emphasize that Notch4 gene is abnormally overexpressed in CRC and is a prospective biomarker for survival of CRC patients [39]. We observed overexpression of Notch4 in 56% of colorectal cancers. In our cohort, Notch4 expression was significantly higher in colorectal cancers than normal (*p*=0.0046) as well as adenoma tissues (*p*=0.036), suggesting its involvement in CRC progression. Furthermore, in our analysis, with the TCGA dataset, Notch4 overexpression was found to be drastically associated with poor survival (*p*=0.0275).

Han and coauthors demonstrated overexpression of Hey1 in CRC tissues and cell lines and attributed it to downregulation of miR-769 that directly targets Hey1 [40]. Furthermore, they found that the overexpression of this downstream effector of the Notch signaling pathway was significantly higher in advanced stage CRCs. Aberrant regulation of Hey1 levels through miR-769 leads to deregulation of several biological processes such as increased cell proliferation and invasion in colorectal cancers [40]. Our data reveal that Hey1 was overexpressed ≥2-fold in 24% of CRCs. Additionally, the Hey1 target gene of Notch signaling was significantly associated with poor survival (*p*=0.0394). Overall, our data demonstrated that deregulation of Notch signaling was detected in 56% of adenomas and 86% of CRCs in our cohort. Though our sample size is small and results need to be interpreted cautiously, activation of the Notch pathway in colorectal cancers versus adenomas is significantly different (*p*=0.0431), implying it to be activated in the progression from adenomas to CRCs. Since we did not observe significant difference in the expression of any of the examined genes between early-stage and late-stage cancers, it would be interesting to demonstrate whether the Notch pathway gets activated during the progression from colorectal adenomas to cancers in a larger cohort. To our knowledge, this is the first study investigating the mRNA expression of 13 important Notch signaling members that include 4 receptors, 5 ligands, and 4 target genes in adenomas and CRCs using highly specific TaqMan assays in Saudi Arabian patients.

In conclusion, our study emphasizes a significant role of the Notch signaling pathway in colorectal cancers and suggests its involvement in prognosis. Hence, targeting this pathway either alone or in combination with other drugs may be a promising strategy in the management of colorectal cancers for better patients' survival outcome.

## Figures and Tables

**Table 1:** TaqMan assay IDs for Notch pathway genes.

No.	Gene	Assay ID
1	Notch1	Hs01062014_m1
2	Notch2	Hs01050702_m1
3	Notch3	Hs01128541_m1
4	Notch4	Hs00965889_m1
5	Jag1	Hs01070032_m1
6	Jag2	Hs00171432_m1
7	Dll1	Hs00194509_m1
8	Dll3	Hs01085096_m1
9	Dll4	Hs00184092_m1
10	Hes1	Hs00172878_m1
11	Hes5	Hs01387463_g1
12	Hey1	Hs01114113_m1
13	Hey2	Hs00232622_m1
14	GAPDH	Hs02758991_g1

## Data Availability

All data used to support the findings of this study are included within the article.

## References

[1] Bray F., Ferlay J., Soerjomataram I., Siegel R. L., Torre L. A., Jemal A. (2018). Global cancer statistics 2018: GLOBOCAN estimates of incidence and mortality worldwide for 36 cancers in 185 countries. *CA: A Cancer Journal for Clinicians*.

[2] Ferlay J., Ervik M., Lam F. (2018). *Cancer Today. Lyon, France: International Agency for Research on Cancer*.

[3] Vatandoust S., Price T. J., Karapetis C. S. (2015). Colorectal cancer: metastases to a single organ. *World Journal of Gastroenterology*.

[4] Annett S., Robson T. (2018). Targeting cancer stem cells in the clinic: current status and perspectives. *Pharmacology & Therapeutics*.

[5] Sonbol M., Ahn D., Bekaii-Saab T. (2019). Therapeutic targeting strategies of cancer stem cells in gastrointestinal malignancies. *Biomedicines*.

[6] Morgan T. H. (1917). The theory of the gene. *The American Naturalist*.

[7] Lai E. C. (2004). Notch signaling: control of cell communication and cell fate. *Development*.

[8] Weng A. P., Ferrando A. A., Lee W. (2004). Activating mutations of NOTCH1 in human T cell acute lymphoblastic leukemia. *Science*.

[9] Tao J., Chen S., Lee B. (2010). Alteration of notch signaling in skeletal development and disease. *Annals of the New York Academy of Sciences*.

[10] Louvi A., Artavanis-Tsakonas S. (2012). Notch and disease: a growing field. *Seminars in Cell & Developmental Biology*.

[11] Vazquez-Ulloa E., Lizano M., Sjöqvist M., Olmedo-Nieva L., Contreras-Paredes A. (2018). Deregulation of the notch pathway as a common road in viral carcinogenesis. *Reviews in Medical Virology*.

[12] Chu D., Zhang Z., Zhou Y. (2011). Notch1 and notch2 have opposite prognostic effects on patients with colorectal cancer. *Annals of Oncology*.

[13] Vinson K. E., George D. C., Fender A. W., Bertrand F. E., Sigounas G. (2016). The notch pathway in colorectal cancer. *International Journal of Cancer*.

[14] Lian H., Jia X., Shi N. (2018). Notch signaling promotes serrated neoplasia pathway in colorectal cancer through epigenetic modification of EPHB2 and EPHB4. *Cancer Management and Research*.

[15] Sikandar S. S., Pate K. T., Anderson S. (2010). Notch signaling is required for formation and self-renewal of tumor-initiating cells and for repression of secretory cell differentiation in colon cancer. *Cancer Research*.

[16] Nwabo K. A. H., Takam K. P., Tagne S. R. (2017). Developmental pathways associated with cancer metastasis: notch, wnt, and hedgehog. *Cancer Biology & Medicine*.

[17] Artavanis-Tsakonas S., Rand M. D., Lake R. J. (1999). Notch signaling: cell fate control and signal integration in development. *Science*.

[18] Alanazi I. O., Khan Z., Bulut (2018). Endocrine and cell surface receptor signaling in breast carcinogenesis. *Breast Cancer and Surgery*.

[19] Piazzi G., Bazzoli F., Ricciardiello L. (2012). Epigenetic silencing of notch signaling in gastrointestinal cancers. *Cell Cycle*.

[20] Wang Z., Li Y., Sarkar F. H. (2010). Notch signaling proteins: legitimate targets for cancer therapy. *Current Protein & Peptide Science*.

[21] Radtke F., Clevers H. (2005). Self-renewal and cancer of the gut: two sides of a coin. *Science*.

[22] Reedijk M., Odorcic S., Zhang H. (2008). Activation of notch signaling in human colon adenocarcinoma. *International Journal of Oncology*.

[23] Fre S., Pallavi S. K., Huyghe M. (2009). Notch and wnt signals cooperatively control cell proliferation and tumorigenesis in the intestine. *Proceedings of the National Academy of Sciences*.

[24] Akiyoshi T., Nakamura M., Yanai K. (2008). *γ*-Secretase inhibitors enhance taxane-induced mitotic arrest and apoptosis in colon cancer cells. *Gastroenterology*.

[25] Lee S. H., Jeong E. G., Yoo N. J., Lee S. H. (2007). Mutational analysis of notch 1, 2, 3 and 4 genes in common solid cancers and acute leukemias. *Acta Pathologica, Microbiologica, et Immunologica Scandinavica*.

[26] Mutvei A. P., Fredlund E., Lendahl U. (2015). Frequency and distribution of Notch mutations in tumor cell lines. *BMC Cancer*.

[27] Brijwani N., Jain M., Dhandapani M. (2017). Rationally co-targeting divergent pathways in KRAS wild-type colorectal cancers by CANscript technology reveals tumor dependence on notch and Erbb2. *Scientific Reports*.

[28] Fischer M., Yen W.-C., Kapoun A. M. (2011). Anti-DLL4 inhibits growth and reduces tumor-initiating cell frequency in colorectal tumors with oncogenic KRAS mutations. *Cancer Research*.

[29] Jia X., Wang W., Xu Z. (2016). A humanized anti-DLL4 antibody promotes dysfunctional angiogenesis and inhibits breast tumor growth. *Scientific Reports*.

[30] Ambrogio C., Gómez-López G., Falcone M. (2016). Combined inhibition of DDR1 and Notch signaling is a therapeutic strategy for KRAS-driven lung adenocarcinoma. *Nature Medicine*.

[31] Mullendore M. E., Koorstra J.-B., Li Y.-M. (2009). Ligand-dependent notch signaling is involved in tumor initiation and tumor maintenance in pancreatic cancer. *Clinical Cancer Research*.

[32] Yamaguchi N., Oyama T., Ito E. (2008). Notch3 signaling pathway plays crucial roles in the proliferation of ErbB2-negative human breast cancer cells. *Cancer Research*.

[33] Bernasconi-Elias P., Hu T., Jenkins D. (2016). Characterization of activating mutations of notch3 in T-cell acute lymphoblastic leukemia and anti-leukemic activity of notch3 inhibitory antibodies. *Oncogene*.

[34] Park J. T., Li M., Nakayama K. (2006). Notch3 gene amplification in ovarian cancer. *Cancer Research*.

[35] Rallis G., Koletsa T., Saridaki Z. (2019). Association of notch and hedgehog pathway activation with prognosis in early-stage colorectal cancer. *Anticancer Research*.

[36] Pasto A., Serafin V., Pilotto G. (2014). Notch3 signaling regulates MUSASHI-1 expression in metastatic colorectal cancer cells. *Cancer Research*.

[37] Ozawa T., Kazama S., Akiyoshi T. (2014). Nuclear notch3 expression is associated with tumor recurrence in patients with stage II and III colorectal cancer. *Annals of Surgical Oncology*.

[38] Serafin V., Persano L., Moserle L. (2011). Notch3 signalling promotes tumour growth in colorectal cancer. *The Journal of Pathology*.

[39] Wu G., Chen Z., Li J. (2018). Notch4 is a novel prognostic marker that correlates with colorectal cancer progression and prognosis. *Journal of Cancer*.

[40] Han C., Song Y., Lian C. (2018). MiR-769 inhibits colorectal cancer cell proliferation and invasion by targeting HEY1. *Medical Science Monitor*.

